# Why, when and how to investigate primary ciliary dyskinesia in adult patients with bronchiectasis

**DOI:** 10.1186/s40248-018-0143-6

**Published:** 2018-08-09

**Authors:** Martina Contarini, Amelia Shoemark, Jessica Rademacher, Simon Finch, Andrea Gramegna, Michele Gaffuri, Luca Roncoroni, Manuela Seia, Felix C. Ringshausen, Tobias Welte, Francesco Blasi, Stefano Aliberti, James D. Chalmers

**Affiliations:** 1 Department of Pathophysiology and Transplantation, University of Milan, Internal Medicine Department, Respiratory unit and Adult Cystic Fibrosis Center, Fondazione IRCCS Ca’ Granda Ospedale Maggiore Policlinico, Via Francesco Sforza 35, 20122 Milan, Italy; 2 Division of Molecular and Clinical Medicine, University of Dundee, Ninewells Hospital and Medical School, Dundee, UK; 3 Department of Respiratory Medicine, Hannover Medical School and German Center for Lung Research (DZL), Hannover, Germany; 4 Department of Otolaryngology and Head and Neck Surgery, Fondazione IRCCS Ca’ Granda Ospedale Maggiore Policlinico, Department of Clinical Sciences and Community Health, University of Milan, Milan, Italy; 5 Medical Genetics Laboratory, Fondazione IRCCS Ca’ Granda Ospedale Maggiore Policlinico, Milan, Italy

**Keywords:** Bronchiectasis, Primary ciliary dyskinesia, Adult, Aetiology

## Abstract

Bronchiectasis represents the final pathway of several infectious, genetic, immunologic or allergic disorders. Accurate and prompt identification of the underlying cause is a key recommendation of several international guidelines, in order to tailor treatment appropriately. Primary ciliary dyskinesia (PCD) is a genetic cause of bronchiectasis in which failure of motile cilia leads to poor mucociliary clearance. Due to poor ciliary function in other organs, individuals can suffer from chronic rhinosinusitis, otitis media and infertility.

This paper explores the current literature describing why, when and how to investigate PCD in adult patients with bronchiectasis. We describe the main PCD diagnostic tests and compare the two international PCD diagnostic guidelines. The expensive multi-test diagnostic approach requiring a high level of expertise and specialist equipment, make the multifaceted PCD diagnostic pathway complex. Therefore, the risk of late or missed diagnosis is high and has clinical and research implications.

Defining the number of patients with bronchiectasis due to PCD is complex. To date, few studies outlining the aetiology of adult patients with bronchiectasis conduct screening tests for PCD, but they do differ in their diagnostic approach. Comparison of these studies reveals an estimated PCD prevalence of 1–13% in adults with bronchiectasis and describe patients as younger than their counterparts with moderate impairment of lung function and higher rates of chronic infection with *Pseudomonas aeruginosa*.

Diagnosing PCD has clinical, socioeconomic and psychological implications, which affect patients’ life, including the possibility to have a specific and multidisciplinary team approach in a PCD referral centre, as well as a genetic and fertility counselling and special legal aspects in some countries.

To date no specific treatments for PCD have been approved, standardized diagnostic protocols for PCD and recent diagnostic guidelines will be helpful to accurately define a population on which planning RCT studies to evaluate efficacy, safety and accuracy of PCD specific treatments.

## Background

Bronchiectasis is a chronic respiratory disease characterized by permanent dilatation of the bronchi and associated with a clinical syndrome of cough, sputum production and recurrent respiratory infections, with attributable morbidity and mortality rates [1, 2]. Although epidemiological data are limited, recent literature suggests that bronchiectasis is far from being a rare disease [3]. Different European datasets describe an increasing prevalence of bronchiectasis with current rates estimated at 53 to 566 cases *per* 100,000 inhabitants [4–6]. Bronchiectasis represents the final pathway of several infectious, genetic, immunologic or allergic disorders which helps explain the extreme heterogeneity of the disease [7]. Identifying the underlying cause both accurately and quickly is a key recommendation of several international guidelines, as many aetiologies of bronchiectasis are treatable or have specific prognostic implications [8].

Primary ciliary dyskinesia (PCD) is widely recognized as an aetiology of bronchiectasis not only in children or young adults but also in older patients [9–11]. PCD is a rare disorder with an estimated prevalence of 1:10,000 [12] caused by mutations in more than 30 genes which leads to functional and/or structural defects of motile cilia [13, 14]. Cilia are specialized hair-like motile or non-motile structures, containing nine pairs of microtubules in a peripheral and radial distribution with a central pair of microtubules. Motile ciliated epithelial cells are present in the nasal cavity, paranasal sinuses, middle ear, airways, fallopian tube, cervix, vas deferens and ependyma. Respiratory cilia mediate the propulsion and expulsion of the mucus layer through coordinated movements (mucociliary clearance). PCD-causing mutations result in immotile or ineffective ciliary beating and consequently in abnormal mucociliary clearance and chronic bacterial infection. This inflammatory and infectious process leads to chronic rhinosinusitis and otitis media, progressive airway obstruction, bronchiectasis and ultimately respiratory failure [15]. In addition, failure of effective motile cilia function in the embryonic node leads to situs inversus in ~ 50% patients with PCD. Sperm flagella and cilia of the fallopian tubes share common axonemal structures with motile cilia of the respiratory system, so a proportion of PCD-affected males and females are infertile [16].

## Why is it important to diagnose PCD in adults with bronchiectasis?

Although the optimal series of tests to investigate bronchiectasis aetiology is still under debate, most of the clinical and scientific community underline the need to investigate and diagnose PCD in adults with bronchiectasis because of its clinical, social-economic and psychological implications. From a clinical perspective, PCD is a multi-system disease with extra-respiratory involvement which benefits from a specific, comprehensive and multidisciplinary team approach in a PCD referral centre [1, 17]. A prompt and accurate diagnosis should be provided to patients because a late diagnosis is associated with an impairment of respiratory function as determined by decreased forced expiratory volume in the 1st second (FEV_1_), and increased likelihood of chronic *Pseudomonas aeruginosa* infection [18]. Furthermore, PCD is a hereditary disorder and patients and their families may require referral to genetic and reproductive counselling services [17].

PCD therapeutic approach largely mimics treatment for other chronic respiratory diseases such as cystic fibrosis (CF) and non-CF bronchiectasis. Chest physiotherapy, including adjunct long-term mucoactive treatment like nebulized hypertonic saline, and specific pulmonary rehabilitation programmes are the cornerstones of PCD long-term therapy [19], accompanied by prompt antibiotic courses for exacerbations guided by historical sputum cultures [17]. Moreover, upper airway involvement may require a disease specific approach, including otolaryngology and audiology counselling [17], as it delivers significant symptom burden and puts patients at risk for sinonasal infections and subsequent pulmonary exacerbations. Hearing aids may be indicated in a considerable number of adult PCD patients. Diagnostic nasal sinus lavage for microbiologic sampling may be reasonable, when chronic infection or inflammation of the paranasal sinuses is suspected or when persistent rhinosinusitis is present. Daily sinonasal saline irrigation may be required for the moisturisation of the nasal mucosa.

To date, no specific treatments for PCD are approved. A multi-centre RCT of six-months azithromycin maintenance therapy is currently ongoing [20]. However, our understanding of the molecular biology of PCD is rapidly improving and in the near future we may see different treatment options for this group of patients. An example of this is the current CLEAN-PCD trial testing the VX-371 compound, an inhaled epithelial sodium channel (ENaC) inhibitor, that improves airway hydration and mucociliary clearence blocking the reabsorption of sodium in the airway surface liquid. The CLEAN-PCD trial is an ongoing international, phase II, randomized, double-blind, placebo-controlled study to evaluate the safety and efficacy of VX-371 inhalation with or without oral Ivacaftor in patients with PCD (NCT02871778).

From a socioeconomic point of view, PCD patients have access to free care and proper health insurance issues in some countries and/or might benefit from special legislation. From a psychological perspective, PCD is a disabling chronic disease with implications for patients’ life plans. Most PCD patients, both male and female, have an increased risk of fertility problems which may go undetected for a long time. Therefore, fertility counselling should be included in PCD standard of care and offered to patients when appropriate [16]. Finally, and above all, patients wish to have a diagnosis and know the reason for their symptoms [21].

## When should PCD be investigated in adults with bronchiectasis?

There are no publications which directly address whether routine aetiological investigation protocols provide benefits in terms of morbidity compared to clinically driven investigations or no testing. The recent guidelines published by the European Respiratory Society (ERS) suggest a *minimum* bundle of aetiological tests to be performed in adults with a new diagnosis of bronchiectasis, including differential blood count, serum immunoglobulins and testing for allergic bronchopulmonary aspergillosis [8]. Testing for PCD is limited to patients with clinical features consistent with the disease: persistent productive cough since childhood, nasal polyposis and/or chronic rhinosinusitis, chronic middle ear disease with or without hearing loss, situs anomalies, congenital cardiac defects and a history of neonatal respiratory distress or neonatal intensive care admittance in term infants. These clinical factors are included in the PICADAR score, a simple diagnostic clinical tool to predict whether symptomatic patients have PCD [22]. Most bronchiectasis guidelines agree with this diagnostic approach targeted on patient clinical features, underlining the importance of evaluating a history of symptoms from upper and lower respiratory tract that goes back to childhood [1, 8, 23]. However, some tertiary referral centres with expertise in PCD testing investigate PCD in most of their bronchiectasis patients [24, 25]. A study shows how a full investigation of aetiologies in adults with bronchiectasis allow diagnosis of PCD in patients without a strong history consistent with the disease. In a cohort of 240 people presenting with chronic productive cough and recurrent chest infections, PCD was found in 17 (10%) patients with a mean age of 36 years (36 ± 13) [11]. This suggests that the prevalence of PCD is greater than is currently known and should be suspected also in adults without typical PCD clinical features, in particular as clinical presentation may vary according to age.

## How to diagnose PCD in adults with bronchiectasis?

ERS guidelines propose a combination of tests to diagnose PCD, including nasal nitric oxide, high-speed video analysis, transmission electron microscopy, immunofluorescence and genetics. Due to poor sensitivity and specificity of many of these modalities, multiple tests rather than a hierarchical strategy of testing are suggested [8]. A short description of PCD diagnostic tests is presented below.

### Clinical presentation

#### PICADAR and modified PICADAR

PICADAR is a simple diagnostic clinical tool to predict whether patients with persistent productive cough are likely to have PCD [22]. It has seven predictive parameters (full-term gestation, neonatal chest symptoms, neonatal intensive care admittance, situs inversus, congenital cardiac defect, chronic rhinitis and ear symptoms) and each contributes a weighted score (1–4) to give a maximum of 14 points. The best discriminative cut-off of the score is 5 points, with a sensitivity of 90% and a specificity of 75%. PICADAR has a good accuracy and has already been validated internally and externally, with an Area Under the Curve of 0.91 and 0.87, respectively. However, most adults do not remember their gestational age or what happened immediately after their birth. Therefore, a modified PICADAR has been recently created to overcome this problem: “gestational age” is omitted and “neonatal chest symptoms” and “admittance to a neonatal unit” are combined to a more general “neonatal respiratory distress” [25]. Each parameter contributes again with 1 to 4 points to the total score, but the best discriminate cut-off is 2, with a sensitivity of 100% and a specificity of 89%. This modified score requires further prospective testing in adult cohorts.

Although both scores have easy and quick items to compute and could be used to decide which patients to refer to further PCD tests, they cannot be used in isolation, because of their limited sensitivity and specificity.

In summary, suspicion based upon history and clinical presentation is essential. Additional historical and clinical features commonly observed in subjects with PCD include parental consanguinity, pectus excavatum and scoliosis [26, 27]. Chest high-resolution computer tomography (HRCT) features can also be helpful in raising PCD suspicion. Typical findings are the characteristic distribution, extent and severity (predominantly middle and lower lobe involvement) the presence of mucus plugging, tree in bud phenomenon, atelectasis, history of middle or lower lobe resection and situs inversus [28].

#### Nasal nitric oxide (nNO)

Measurement of nNO is one of the first steps in the PCD diagnostic flowchart, because it is non-invasive, affordable and relatively easy to perform, especially thanks to the increasing availability of electrochemical portable analyzers [29]. Therefore, nNO superseded saccharin test, a previous test of nasal mucociliary clearance, no longer advocated because it is not suitable for small children and is prone to false positive and negative results [30].

Nasal NO is used in the PCD diagnostic algorithm because nNO levels are extremely low in most patients with PCD when compared to healthy and disease controls [31]. The reason behind this low nNO is unknown. Many hypotheses have been proposed so far, including a reduced biosynthesis of nitric oxide (NO) by the airway epithelium [32]; an increased breakdown of NO to metabolites within respiratory cells or by denitrifying bacteria [33]; a reduced storage capacity of NO in the paranasal sinuses [34] and NO trapped in the obstructive paranasal sinuses [35], but none of them is unanimously accepted.

There is also no consensus on the most appropriate technique for velum closure, analyser sampling rate (to which the measurement is sensitive) or what nNO threshold constitutes a positive or negative cut-off [36]. Leigh and colleagues developed a threshold of 77 nL/min using a conversion calculation which is reported to account for analyser differences with a sensitivity of 99% and a specificity of 75% [37]; Beydon and co-workers used a cut-off of 82 nL/min, reporting a sensitivity of 91% and a specificity of 86% [38], whereas Jackson and colleagues provided a sensitivity and specificity of 91 and 96%, respectively, with a threshold of 30 nL/min [39]. To date, 77 nL/min is one the most used nNO cut-off and has been accepted as a diagnostic criterion for PCD by the North America PCD Foundation [17].

Many conditions though reduce nNO levels, including nasal polyps [40], chronic sinusitis [41], diffuse panbronchiolitis [42], cystic fibrosis (CF) [43], HIV infection [44] and smoking [45]. In addition, PCD genotypes associated with normal nNO levels have been increasingly reported [46, 47]. Therefore, although the value of nNO as part of the diagnostic algorithm for PCD is clear, its lack of specificity prevents it from being used in isolation to rule PCD in or out [36]. So, patient’s clinical history should be considered in conjunction with nNO results when deciding whether to proceed with a nasal brush biopsy [48].

#### High-speed video analysis (HSVA)

Ciliary function can be analyzed ex vivo by the assessment of respiratory ciliary activity in epithelium obtained by cytology brush from the nose or bronchus. A camera attached to the microscope records at high speeds (120–500 frames per second – fps -) and video can be replayed slower (30–60 fps) to measure ciliary beat frequency (CBF) and review ciliary beat pattern (CBP) [49]. However, this requires considerable expertise, and there is no consensus on appropriate cell processing and method of ciliary assessment. Therefore, standardized protocols and thresholds need to be developed for ex vivo analysis of CBF and CBP. Ciliated cells can be observed immediately after sampling and again after a period of culturing to differentiate PCD from secondary dyskinesia, a ciliary defect caused by infection and inflammation of the airways [50]. Secondary ciliary dyskinesia can affect CBF (low CBF) and CBP (poor coordination of ciliary beating, lack of full sweep and mucus impedance) and therefore repeating the analysis following cell culture in the presence of antibiotics is sometimes recommended to differentiate it from a primary defect [36].

#### Transmission electron microscopy (TEM)

The evaluation of ciliary ultrastructural defects is a key part of the diagnostic work-up. TEM is a highly specific test, considered for many years the “gold standard” diagnostic test for PCD, because it can detect lack of dynein arms [51], disorganizations of microtubular doublets [52] or loss of the central microtubular pair [53]. However, TEM should not be used in isolation to exclude a PCD diagnosis, because standardized protocols and consensus on terminology, especially regarding the number and proportion of cilia required to make a diagnosis, are lacking and 15–30% of patients with PCD have apparently normal ciliary ultrastructure, for example those with a pathogenic variant in DNAH11 [36, 54]. It is important to underline that acquired defects can be associated with transient ultrastructural abnormalities (disrupted membranes, compound cilia, additional tubules and axonemal blebs) [50], so repeating TEM after cell culture may be helpful in equivocal cases.

#### Immunofluorescence

Immunofluorescence (IF) can help confirm PCD using fluorescent antibodies to stain ciliated respiratory epithelium obtained by nasal brushing. IF can detect the absence of key ciliary proteins such as the DNAH5 heavy chain of the outer dynein arm [55]. IF may identify some cases where TEM is apparently normal or subtly abnormal [56, 57] and it is cheaper and quicker than TEM, but, although its accuracy has recently been determined, its sensitivity remains limited [58]. Many PCD mutations and structural cilia proteins have not been identified yet and false negative results may occur, but gene discovery and antibody development are likely to improve immunofluorescence sensitivity and technique.

#### Genetics

Genetic testing for disease-causing mutations can be performed in patients suspected of having PCD to confirm the diagnosis such as those with normal ultrastructure by TEM or in already confirmed PCD patients to provide a genetic diagnosis. Non-ambiguous bi-allelic autosomal mutations or hemizygous X-linked mutations are identified in 50–75% of cases [59]. To date, more than 1000 disease causing variants across more than 35 genes have been described and the most common are in the dynein heavy chains DNAH5 and DNAH11 [60]. However, not all PCD mutations are known and accuracy of genetic testing is still lacking.

### Diagnostic testing guidelines

Currently there are two specific PCD guidelines that propose diagnostic criteria and describe diagnostic tests: the guidelines published by ERS [36] and the consensus recommendations published by the North American PCD Foundation [17]. They differ in many aspects. First of all, ERS guidelines are evidence-based guidelines, whereas the American Genetic Disorders of Mucociliary Clearance Consortium (GDMCC) created a consensus statement. The American recommendations distinguish PCD diagnostic criteria by age and define PCD diagnosis according to the presence of two or more major clinical criteria (unexplained neonatal respiratory distress, any organ laterality defects, chronic wet cough and rhinosinusitis since childhood) plus at least one positive diagnostic test (nNO, HSVA, TEM and genetics). ERS, instead, do not distinguish PCD diagnostic criteria by age, but provide a diagnostic algorithm with a step-wise approach ending in different outcomes: PCD positive, PCD highly likely and PCD extremely unlikely.

The two publications differ also in some diagnostic test aspects. The American statement recommends 77 nL/min as nNO threshold, whereas ERS guidelines cannot find a consensus on the appropriate nNO cut-off. ERS guidelines recommend the use of HSVA before and after cellular culture as an important diagnostic tool, while the American statement, although recognizing its importance, underlines the fact that few centres have expertise to perform these tests.

The ERS guidelines and the American statement share also some similarities: both include infants and adults; both could not recommend IF as a diagnostic tool because studies were lacking, although these studies have now been performed [58] and both advise against the use of TEM in isolation to exclude PCD because some patients with PCD show apparently normal ultrastructure.

The comparison between the two publications is summarized in Table 1.

**Table 1:** A comparison between European Respiratory Society (ERS) and North American primary ciliary dyskinesia (PCD) Foundation guidelines

	ERS 2017 [36]	North American PCD Foundation 2016 [17]
Structure	Evidence-based guidelines	Consensus statement
Patients included	Infants and adults	Infants and adults
Diagnostic criteria distinguished by age	Not done	Newborns (0–1 month) Children (1 month – 5 years) Children 5–18 years and adults
Diagnostic criteria	A diagnostic algorithm is proposed	Two major clinical criteria PLUS at least one diagnostic test
Diagnostic outcome	PCD positive, PCD highly likely and PCD extremely unlikely	PCD positive and PCD negative
nNO	No consensus on appropriate thresholds	< 77 nL/min on 2 occasions, > 2 months apart, with CF excluded
HSVA	Several European centres employ HSVA due to high expertise	No American centres can reliably perform HSVA due to lack of expertise
Cell culture	Recommended to improve accuracy of HSVA and TEM to rule out a false positive diagnosis or support a highly likely diagnosis	Not mentioned
IF	Not included in the diagnostic algorithm due to lack of studies at time of guideline	Not included in the diagnostic criteria due to lack of studies at time of guideline
TEM	Can be used to confirm a diagnosis but advise against TEM in isolation to exclude PCD because some patients with PCD have apparently normal ultrastructure	Can be used to confirm a diagnosis but advise against TEM in isolation to exclude PCD because some patients with PCD have apparently normal ultrastructure
Genotyping	Can be used to confirm a diagnosis but cannot be used to exclude a diagnosis because evidence on sensitivity and specificity lacks	Can be used to confirm a diagnosis but cannot be used to exclude a diagnosis because evidence on sensitivity and specificity lacks

*PCD* Primary ciliary dyskinesia, *nNO* Nasal nitric oxide, *CF* Cystic fibrosis, *HSVA* High-speed video analysis, *IF* Immunofluorescence, *TEM* Transmission electron microscopy

## Prevalence of PCD in adults with bronchiectasis

Defining the prevalence of PCD in adults with bronchiectasis is challenging.

Some epidemiological information comes from papers that, despite having other aims, describe adult bronchiectasis populations and briefly their aetiologies, including ciliary dysfunction [61–68].

Other studies are focused on defining the demographic characteristics and identifying the aetiologies of patients with bronchiectasis, but do not describe the criteria used to diagnose PCD [69–73], or do not describe any PCD patients in their cohorts [74–76]. To our knowledge, two retrospective studies and ten prospective studies outline the aetiology of adults with bronchiectasis and conduct screening tests for PCD [11, 24, 77–86]. As regards the PCD diagnostic approach though, they differ in many aspects. Most studies use clinical features and history of upper and lower respiratory symptoms and/or infertility as a PCD screening method for further testing [24, 77, 78, 81–83, 85, 86], while one study performs chest high-resolution computed tomography (HRCT) to find diffuse bronchiectasis, considering it a characteristic to investigate for PCD [79].

The studies differ also in the selection of further tests: saccharin test alone [83]; saccharin test and/or nNO [24, 85]; saccharin test, NO test or nasal mucociliary clearance by sero-albumin [86]. Finally, TEM [79, 82, 85], ciliary function [81] or referral patients to a PCD centre [24, 82] are chosen as diagnostic approach to confirm PCD.

Three studies test all adult bronchiectasis patients for PCD, but again they select different assessments: saccharin test [84], saccharin test plus nNO [11] and light microscopy for the CBF [80] as first steps; TEM [80], HSVA plus TEM [11] and Kartagener’s features [84] to confirm PCD diagnosis.

In all the mentioned studies PCD prevalence varies from 1 to 13% in adults with bronchiectasis, but different screening tools and diagnostic tests make this assessment quite difficult to interpret. It will be of interest to see the effect of the recently published diagnostic guidelines on the approach to such studies going forward.

Some of the prospective studies describe the resultant PCD cohort. PCD patients who have been identified are younger than other bronchiectasis patients [11, 24, 84, 86], without differences in gender prevalence and non-smokers [24, 86]. They have a higher prevalence of bilateral bronchiectasis (48.3%) and a moderate impairment of FEV_1_ (67.1% ± 24.2) [86]. PCD patients have higher rates of chronic infection with *P. aeruginosa* (45%) and are treated with chronic inhaled antibiotic more frequently (39.6%) when compared with other patients with bronchiectasis [86].

The comparison between the publications is summarized in Table 2.

**Table 2:** Comparison between studies evaluating aetiologies of bronchiectasis in adult patients

Paper	Type and site of study	Patients involved	Patients screened for PCD	PCD diagnosed	Screening tests	Diagnosis tests	Features of PCD population
Amorim, 2015 [77]	Monocentric, Retrospective, Cohort study Portugal	202	5 with history of infertility	1 (0.5%)	Semen analysis	Not mentioned	Not mentioned
Kadowaki, 2015 [78]	Monocentric, Retrospective, Cohort study Japan	147	147	2 (1%)	Kartagener’s features	Kartagener’s features	Not mentioned
Verra, 1991 [79]	Monocentric, Prospective, Cohort study France	53	38 with diffuse bronchiectasis	5 (13%)	Chest HRCT	TEM	Prevalence in North African patients 36% Prevalence in European patients 4%
Pasteur, 2000 [80]	Monocentric, Prospective, Cohort study United Kingdom	150	150	1 (0.6%)	Light microscopy with CBF	TEM	Not mentioned
King, 2006 [81]	Monocentric, Prospective, Cross-sectional Australia	103	103	1 (1%)	Unexplained infertility	Ciliary function	Not mentioned
Shoemark, 2007 [11]	Monocentric, Prospective, Cohort study United Kingdom	240	240	17 (10%) 12 PCD positive 5 PCD likely	Saccharin test nNO	Light microscopy TEM	Age 36 ± 13
Anwar, 2013 [82]	Two centres Prospective, Cohort study United Kingdom	189	189	2 (1%)	History of upper and lower respiratory symptoms and/or infertility	Referral to specialized PCD centre	Not mentioned
Qian, 2015 [83]	Multi-centre, Prospective, Cohort study China (Chinese Han ethnicity)	476	71 with history of upper and lower respiratory symptoms or Kartagener’s features	4 (0.9%)	Saccharin test	TEM	Not mentioned
Lonni, 2015 [24]	Multicentre, International, Prospective, Cohort study Italy United Kingdom Spain Greece Belgium Ireland	1258	Not mentioned	21 (1.7%)	History of upper and lower respiratory symptoms Saccharin test nNO	Referral to specialized PCD centre	Younger than 50 years Non-smokers Mild disease 11 (52.4%) Moderate 8 (38.1%) Severe 2 (9.5%)
Guan, 2015 [84]	Multicentre, Prospective, Cohort study China	148	148	2 (1.4%)	Saccharin test	Kartagener’s features	Younger than other bronchiectasis patients
Dimakou, 2016 [85]	Monocentric, Prospective, Cohort study Greece	277	32 with history of respiratory distress syndrome after birth, of upper and lower respiratory symptoms since childhood and/or infertility	12 (4%)	Saccharin test nNO	TEM	Not mentioned
Olveira, 2017 [86]	Multi-centre, Prospective, Cross-sectional Spain	2047	Not mentioned	60 (2.9%)	Clinical features Saccharin test Seroalbumin NO (not specified if FE_NO_ or nNO)	TEM	Age 42.9 ± 8.8 Age at diagnosis 22.1 ± 18.1 Male 29 (48.3%) Smokers 14 (23.3%) BMI 23.8 ± 6.1 FEV_1_ (%) 67.1 ± 24.2 Chronic Infection Pa 27 (45%) Inhaled ATB 21 (39.6%) Bilateral bronchiectasis 29 (48.3%)

Features of PCD populations are presented as mean ± SD or number (%)

*PCD* Primary ciliary dyskinesia, *HRCT* High-resolution computed tomography, *nNO* Nasal nitric oxide, *HSVA* High-speed video analysis, *TEM* Transmission electron microscopy, *CBF* Ciliary beat frequency, *FE*_*NO*_ Orally exhaled nitric oxide, *SD* Standard deviation

PCD published studies are few with low numbers of patients involved. Recently, two large international registries (iPCD Cohort and International PCD registry) have been created, including over 3000 PCD patients [9, 10]. Although these registries have the strength of assessing differences in PCD characteristics and management between countries and ethnic groups, they have also some limitations in defining what a positive diagnosis of PCD is. They also have a predominance of paediatric patients, suggesting adults with PCD are being neglected from a research perspective.

## Conclusion

Physicians treating bronchiectasis patients should suspect the diagnosis of PCD in adults, in order to offer appropriate care. The requirement for a multi-test diagnostic approach where some tests are very expensive, some requiring a high level of expertise and specialist equipment, make the multifaceted PCD diagnostic pathway complex. Therefore, the risk of heterogeneous, late or missed diagnosis is high [9, 18] and has clinical and research implications. Standard diagnostic protocols for PCD will be helpful to accurately define a population on which planning RCT studies to evaluate efficacy, safety and accuracy of treatments.
